# Relationship between malaria vector survival, infectivity and insecticide treated net use in western Kenya

**DOI:** 10.21203/rs.3.rs-4090984/v1

**Published:** 2024-03-18

**Authors:** Lucy Abel, Emma Kimachas, Evans Omollo, Erick Nalianya, Tabitha Chepkwony, Joseph Kipkoech, Mark Amunga, Aggrey Wekesa, Jane Namae, Samuel Kahindi, Judith Mangeni, Zena Lapp, Christine Markwalter, Steve M. Taylor, Andrew Obala, Wendy Prudhomme O’Meara

**Affiliations:** AMPATH; AMPATH; Duke Global INC; Duke Global INC; AMPATH; AMPATH; AMPATH; Duke Global INC; Moi University School of Medicine; Pwani University; Moi University School of Public Health; Duke University NC; Duke University NC; Duke University NC; Moi University School of Medicine; Duke University NC

**Keywords:** Anopheles, Survival, blood meal, Infection rates, ITNs

## Abstract

**Background:**

Much effort and resources have been invested to control malaria transmission in Sub-Saharan Africa, but it remains a major public health problem. For the disease to be transmitted from one person to another, the female *Anopheles* vector must survive 10–14 days following an infective bite for the *Plasmodium*gametocytes to develop into infectious sporozoites which can be transmitted to the next person during a bloodmeal. The goal of this investigation was to assess factors associated with wild-caught Anopheles survival and infection following host-seeking and indoor resting.

**Methods:**

The study was conducted in a longitudinal cohort of 75 households in 5 villages including a total of 755 household members in Bungoma County, Kenya. Monthly adult mosquito collection was conducted by attenuated aspiration in all the enrolled households, and the mosquitoes were reared in the insectary for 7 days. The daily mortality rate was determined through day 7, and all the mosquitoes were morphologically identified. Female *Anopheline* mosquitoes were dissected, and species-level members of the *Anopheles gambiae* complex were resolved by molecular methods. The abdomen for all samples were processed for *P. falciparum* detection by PCR.

**Results:**

Within a period of 25 months, the total number of culex and *Anopheles* mosquitoes collected indoors were 12,843 and 712 respectively. *Anopheles gambiae*and *Anopheles funestus* were the major vectors though their population varied between different villages. 61.2% (n=436/712) of the *Anopheles* species survived up to day 7 with the lowest mortality rate recorded on day 5 of captivity. The survival rate also varied between the different *Anophelesspecies*. 683 of 712 mosquito abdomens were tested for *P. falciparum*detection and 7.8% (53/683) tested positive for *P. falciparum* with *An. funestus* having **a** higher (10%) prevalence than *An. gambaie s.s*.(6.0%, p=0.095, Pearson Chi square test). The proportion of household members sleeping under a bednet the night before mosquito collection varied across time and village. *An. funestus* survival times were refractory to household ITN coverage and *An. gambaie s.s*. survival was reduced only under very high (>95%) ITN coverage.

**Conclusion:**

Despite ITN coverage, mosquitoes still acquired bloodmeals and *P. falciparum* infections. Survival differed across species and was inversely correlated with high ITN exposure in the household, but not oocyst development.

## INTRODUCTION

Malaria transmission continues across most of sub-Saharan Africa despite the scale up of effective vector control methods. Anopheles mosquitoes which transmit malaria must survive 10–14 days following an infectious bloodmeal in order for the parasite to complete development to the infectious stage (sporozoites)[1]. Therefore, transmission has been predicted to be highly sensitive to the longevity of the vector. [2]Vector control tools such as insecticide treated nets (ITNs) and indoor residual spraying (IRS) are thought to cause mortality among host-seeking or resting mosquitoes as well as reduce the lifespan of the vector. Such interventions should be effective in reducing transmission if they shorten the average lifespan of the vector population [3] and reduce the number of vectors that survive the extrinsic incubation period.

Malaria transmission is highly dependent on mosquito longevity, which is difficult to measure under natural conditions. Standardized assays to estimate vector sensitivity to specific insecticides can only determine chemical resistance and typically observe survival over short time periods after controlled exposures, therefore failing to capture more subtle effects on mosquito survival [4]. However, studying the longevity of malaria vectors after natural exposure to ITNs during host-seeking is important in understanding the efficacy of the ITNs.

This study was conducted to measure wild-caught *Anopheles* survival and infection following host-seeking and indoor resting. The study was conducted within a household-based longitudinal cohort study in Western Kenya, where malaria transmission is seasonal and primarily transmitted by *An gambiae* and *An funestus*. Resting mosquitoes were aspirated from inside homes and reared for seven days to investigate the relationship between household ITN use, human infectivity to mosquitoes, and mosquito survival. Looking closely at these interdependent factors enable us to understand if there is correlation between mosquito infection, mosquito survival and ITN use in western Kenya.

## METHODS

### Study area and cohort

The study was carried out in Webuye East and West sub counties located in western Kenya. The sub counties are rural, and most families engage in small-scale farming and animal husbandry. Malaria transmission is moderate and perennial with 2 seasonal peaks after the long rains (May–June) and the short rains (Sept–October) although the timing and intensity of the transmission peaks can vary from year to year.

From January 2020 we followed a cohort of 755 people aged 1 to 100 years living in 75 households in a rural setting in Webuye, western Kenya who regularly slept in the household. The cohort was assembled in five villages with moderate to high malaria transmission using radial sampling of 15 households per village, beginning with a randomly selected household in each village. During the study period, seven households were replaced, three which moved and four that withdrew.

Face-to-face interviews were conducted by field staff during monthly visits to record information about who slept in the household and bednet use. This included questions including whether the person slept under a bednet the previous night, what time they typically go to bed and where they spend time in the evening before going to bed. Those with recent overnight travel were also asked if they slept under a bednet during their trip.

### Entomological collections

The study team visited each household once per month between 6.00 a.m. and 8.00 a.m. to collect indoor resting mosquitoes via aspiration with Prokopacks (John W Hock Company). The Prokopacks were fitted with a custom attachment to reduce the aspiration force and decrease damage to mosquitoes during collection. Participants were asked to leave doors and windows closed until the team arrived. Mosquitoes were collected and stored in collection cups inside insulated boxes with 10% sucrose solution dipped in cotton wool attached to each cup with a masking tape for feeding mosquitoes until they were transported to the insectary lab.

In the insectary, we released mosquitoes from a single household into individual enclosed cages in a room maintained at 27 C ± 2 and 80%+10% humidity. They were fed on 10% sucrose solution, which was refreshed every 2 days. Daily mosquito mortalities were recorded from day 0 to day 6. On day 7, all surviving mosquitoes were sacrificed.

Mosquitoes were sorted by genus and sex on the day they died. Female *Anopheles* were imaged under magnification for species identification. After photographing the wings, palp and hind leg, female *Anopheles* mosquitoes were dissected and the head/thorax, abdomen and the wings were stored in separate barcoded tubes packed with desiccant at room temperature. Anopheles were identified to the species based on distinguishable characteristics following the Coetzee key [5]. All species identifications were done by two independent observers blinded to the others’ read. Discordant identifications were reviewed by a senior entomologist.

### Molecular analyses

Members of the Anopheles gambiae complex were distinguished by PCR [6]. Anopheles wing samples were transferred to 96 well plates and extracted using the Hotshot DNA Extraction technique. 50μl of alkaline lysis solution was added to each well and incubated at 95C for 30 mins in a PCR machine. An equal volume (50μl) of neutralizing solution (Tris HCL PH 5.0) was added and stored at −20C. Multiplex species PCR was run on 5μl of extract using primers specific for *Anopheles gambiae sensu stricto* (463bp) and *Anopheles arabiensis* (383bp). Reactions were run on a 1% agarose gel at 100V for 40 minutes. DNA fragments were visualized under UV light using SYBR Safe stain added to the agarose gel.

Anopheles abdomen samples were processed for *P. falciparum* detection using the technique described in [Sumner Nat Com]. Briefly, the mosquito parts were ground using a sterile homogenizer in well labeled microcentrifuge tubes containing 100μl of 10% saponin. The homogenized samples were transferred to a 96-well plate and gDNA extraction done using the simplified chelex extraction method [7]. A duplex real time PCR assay targeting pfr364 and human beta-tubulin was performed by amplifying both targets from the samples along with a set of controls at known densities to detect and quantify *P. falciparum* in each sample.

Mosquitoes which were identified morphologically as An. gambiae s.l. were tested using molecular methods to distinguish An. gambiae s.s. and An. arabiensis. An. gambiae s.l. specimens which did not amplify with primers for An. gambiae s.s. or arabiensis are designated as An gambiae s.l. and are probably another member of that complex.

### Data analysis

Entomological data were recorded on paper forms and then entered into Redcap. Household information was collected and managed using Redcap mobile electronic data capture tools hosted at Duke University [8, 9]. All data were cleaned analyzed using StataSE v17 and visualized using R v4.2.1 (23) in RStudio v2022.12.0 + 353, with the following libraries (tidyverse, dplyr, survminer, ggsurvfit, lubridate)

## RESULTS

Between January 2020 to March 2022, mosquitoes were collected during 25 collection days in 75 households (1875 individual collections) yielding a total of 12,843 female culex and of 712 female anopheles mosquitoes captured indoors and reared in captivity.

### Species distribution

**Figure 1:**
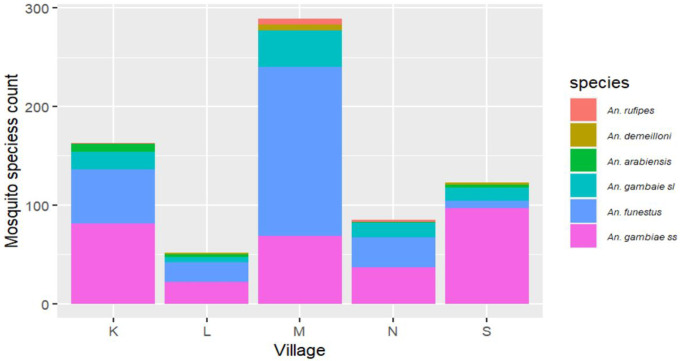
Distribution of *Anopheles* mosquito species by village

*An. gambiae s.s*. (42.9%) and *An. funestus* (39.8%) were the most common vectors, although their relative abundance differed between villages (Table 1, Fig. 1). In one village, the large majority of captured vectors were *An. gambiae* s.s. (Village S, 78.9%, Table 1). In contrast, *An. funestus* was dominant in Maruti (59.2%). In the remaining villages, proportions of *An. funestus* and *An. gambaie ss* were more even, with *An. gambaie ss* slightly outnumbering *An. funestus*. Other mosquito species identified included *An. rufipes 1.3*%), *An. demeilloni* (*1.4*%) and *An. arabiensis* (*2.1*%) which were captured infrequently.

### Female Anopheles survival

**Figure 2:**
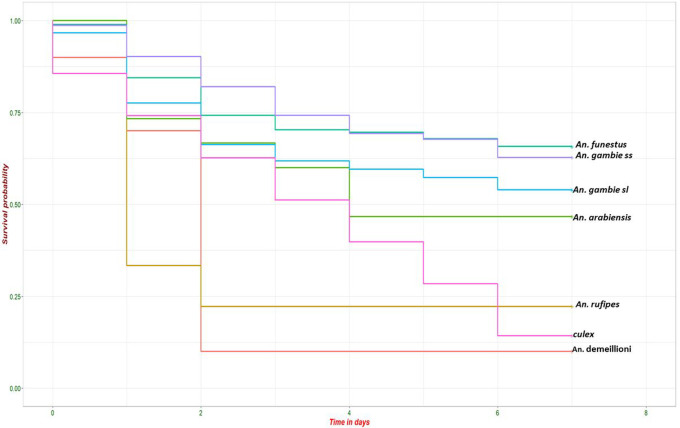
Survival by species from day 0 to day 7.

Of the 712 female anopheles captured and reared, 61.2% (n = 436) survived up to day 7. The lowest mortality rate was recorded on day 5 of captivity. Survivorship to day 7 was slightly different between the vector species (P = 0.0005, Fisher’s Exact test, Fig. 2), with the lowest mortality rates by day 7 observed for *An. gambaie s.s*.(44%) and *An. funestus* (43%). These two vectors had slightly higher mortality in the first few days compared to days 3–7. The minor vectors had lower survival rates, particularly *An. demeilloni* and *An. rufipes* which showed very high mortality within the first two days of capture. Female culex mosquitoes showed consistent daily mortality rates with less than 15% surviving to day 7, unlike the dominant *Anopheles* species whose mortality rate declined after the third day.

### P. falciparum infection and Anopheles survival

683 of 712 *Anopheles* abdomens were tested for the presence of *P. falciparum*. Overall, parasites were detected in 7.8% (53/683) of abdomens. Infection rates differed by species. The P. falciparum prevalence was higher in *An. funestus* (10%) than in *An. gambaie s.s*. (6.0%, p = 0.095, Pearson Chi square test; Table 2).

**Figure 3:**
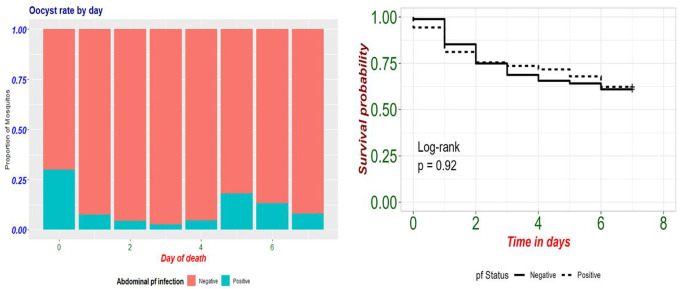
(a) Abdominal *P.falciparum* infection by day of death and (b) daily surviving proportion by abdominal infection status

The abdominal *P. falciparum* prevalence was highest in vectors that died immediately on day 0 (30%, 3/10; Fig. 3a) followed by those that died on day 5 (18.2%, n = 2/11), The *P. falciparum* prevalence was 7.9% (n = 33/417) among vectors surviving to day 7. There was no difference in survival among infected and uninfected anopheles (Fig. 3b, log-rank test, p = 0.58).

### Bednet use and Anopheles survival

**Figure 4:**
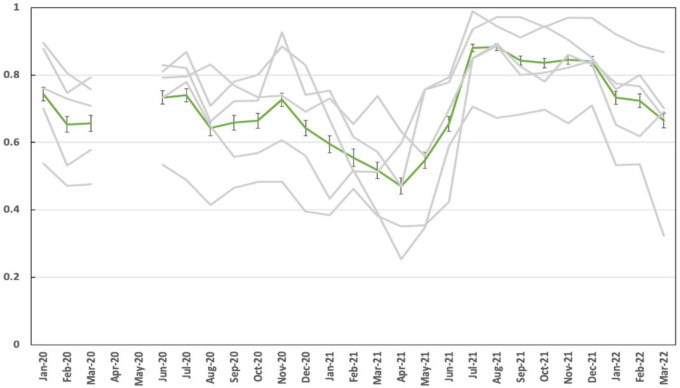
The proportion of people in each of five villages (grey lines) reporting sleeping under a net the night before the survey. The mean and 95% confidence intervals are shown in green. Reported net use rose sharply in June 2021 following a mass net distribution campaign. No data or samples were collected in March and April 2020.

The proportion of household members sleeping under a bednet the night before mosquito collection varied across time and village, ranging from 46–87% (Fig. 4).

**Figure 5:**
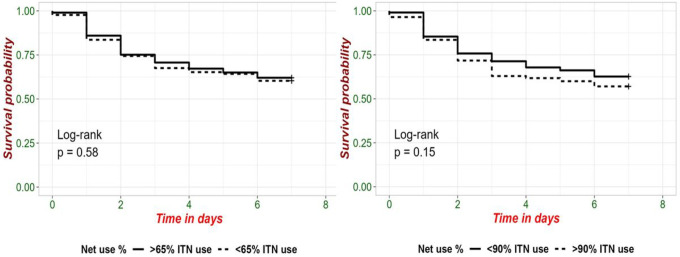
Female *Anophelessurvival* between day 0 and day 7 for mosquitoes in households with higher (dotted line) or lower (solid line) ITN coverage. (a) Households with at least 65% of members sleeping under an ITN the night before collection compared to less than 65% coverage (b) Households with at least 90% of members sleeping under an ITN compared to less than 90% coverage.

We calculated the proportion of household members sleeping under an ITN in each household for each day of mosquito collection and correlated ITN coverage with the survival of *Anopheles* mosquitoes from that household. There was no difference in *Anopheles* survival when comparing vectors collected from households where at least 65% of the members slept under an ITN versus those with lower net coverage. However, when 90% of members slept under an ITN, anopheles collected from those households had lower survival than households with lower coverage, although the comparison did not reach statistical significance (Fig. 5, Log rank test p = 0.15). In households with at least 90% ITN coverage the night before collection, 56.7% of female anopheles survived to day 7 compared to 62.7% in other households.

**Figure 6:**
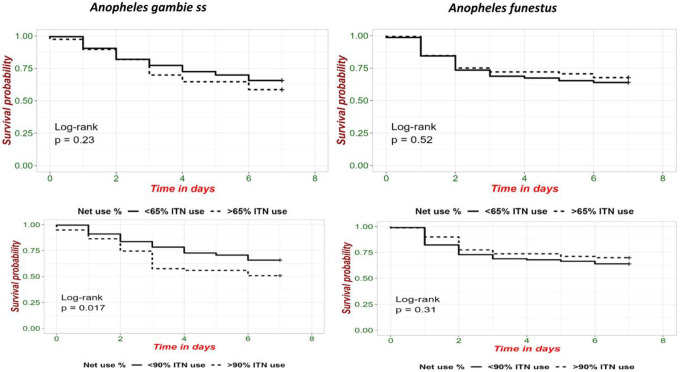
Female *Anopheles* survival between day 0 and day 7 post-collection for mosquitoes collected in households with higher (dotted line) or lower (solid line) ITN coverage. (a,c) *An. gambiae s.s*. (b, d) *An. funestus*

Species-specific survival in households with high or low ITN coverage varied. *An. gambiae s.s*. mosquitoes were more sensitive to ITN exposure and showed greater differences in survival based on ITN coverage. *An. funestus* exhibited much less difference in survival to seven days when captured in households with high ITN coverage compared to *An. gambiae s.s*. (Fig. 6).

## DISCUSSION

In this study we investigated the survival of wild-caught mosquitoes resting in homes in the early morning. These endophilic, likely endophagic, vectors were exposed to differing levels of both ITN use and prevalence of malaria infected hosts [10]. To study the impact of these exposures on wild vector populations, we reared them in cages for seven days and recorded daily mortality. We found that most female anopheles survived to the seventh day. Survival differed across species and was inversely correlated with high ITN exposure in the household, but not oocyst development.

We identified a diverse vector population dominated by two primary vectors, *An. gambiae s.s*. and *An. funestus*. Major vector species had similar survival rates, while minor species exhibited poorer survival following collection. Oocyst infection rates were high for both major vectors, and was nearly 8% in those surviving to day 7. This is similar to what was found in western Kenya closer to Lake Victoria and in our previous work in the study area [11]. If we assume an infection prevalence of 30% in the human hosts [Sumner], uniform biting rates, and that 80% of anopheles have fed in the last 24 hours [10] then we estimate that 1 in 3 bites on an *infected* host must be *infectious* to achieve an 8% oocyst rate.

Regarding the relationship between survivorship and *P. falciparum* oocyst development in malaria vector mosquitoes, we found that infections acquired on or near the day of capture had no effect on survival up to seven days. Very few studies have assessed how infected or uninfected mosquitoes differ in survivorship, especially for wild caught mosquitoes. Most studies have been conducted on mosquitoes reared and infected under laboratory conditions. Significant reduction in survival following infection is limited to studies with combinations of vectors and plasmodium species that are not known to occur naturally [12]. The study by Chege and Beier [13] examined the effect of malaria parasites on the longevity of wild-caught, naturally infected anopheles. The results from their study are consistent with our findings that infection status does not affect survivorship. However, in their study they observed higher survival rates and lower infection rates among *An. funestus* compared to *An. gambaie s.l.*.

Leveraging detailed ITN use data recorded at every mosquito collection time point, we were able to correlate vector survival with ITN coverage at the household level. From our study we found that mosquitoes collected from households that had more than 90% of its members sleeping under a net the night before mosquito collection had a significantly lower survival rate. However, this effect was not observed at lower ITN coverage. In an experimental hut system in Burkina Faso, there was no difference in long-term survival of wild-entry anopheles when they entered a hut with an untreated net or a pyrethroid-treated net [14]. In experimental studies, it has been shown that insecticide-susceptible anopheles can feed across an ITN when the bloodmeal is touching the net. [15] Although feeding time and bloodmeals are smaller, only 15% of fed mosquitoes die in the 24 hours post-feeding indicating that the bloodmeal taken across the net may be protective against mortality. Given that 80% of the anopheles collected in this cohort show evidence of recent feeding [10] feeding success could partially explain the low mortality after foraging in a household with high ITN coverage, although it is unlikely that the majority of available hosts are touching their ITN while sleeping. The observation that feeding reduces mortality from insecticides has been reported elsewhere [16, 17] but whether this is cause (the bloodmeal reduces susceptibility to the chemical) or effect (insects are more successful at feeding if they are less sensitive to the chemical) is difficult to untangle in a natural system such as ours. It is also possible that insects which died quickly after exposure to ITNs were not aspirated from walls and the collected mosquitoes represent a surviving subset of all foraging mosquitoes. Nonetheless, our findings are consistent with epidemiological studies which show that ITNs reduce incidence of malaria in areas with populations of resistant anopheles, but protection is incomplete and transmission persists. [18]

The impact of ITN coverage on survival differed by species. While *An. gambiae s.s*. survival was sensitive to ITN coverage, *An. funestus* survival did not decline with increasing ITN coverage. This could be due to differences in biting behaviors, such as late evening or early morning biting by *An. funestus*, that reduce exposure to ITNs during feeding or it could be due to higher levels of pyretheroid resistance in *An. funestus* compared to *An. gambiae s.s*. which has been reported elsewhere [19]. Overall, the 48-hour mortality rate after foraging and resting in a household with 80 or 90% ITN coverage was less than 25% and differences in survival of *An. gambiae s.s*. exposed to high or low ITN coverage were not noticeable until after day 3. It is unclear whether these small differences in day 7 survival as a function of ITN coverage would reduce overall transmission [3].

Limitations to our study include unknown effects of collection technique on mosquito fitness and survival as well as unknown age and life history of the wild caught mosquitoes. We observed that mosquito vectors had slightly higher mortality in the first few days compared to days 3–7, which might be due to damage during mechanical aspiration of mosquitoes or poor adaptation to cages. Although we reduced the aspiration force of the prokopacks using a bespoke attachment which improved survival, they may still suffer damage during collection. In addition, since it is impossible to determine the ages of the harvested mosquitoes and it is possible that older mosquitoes were more vulnerable to the trauma of aspiration, early mortality may be biased towards older insects. This is supported by the observation that infection rates were higher in mosquitoes that died on the same day they were collected; these mosquitoes must have already survived a minimum of 5–7 days to have been infected on the day of collection. However, since our study was conducted over 24 months, we expect that we collected a representative age distribution of vectors across the study which increases the generalizability of our findings.

In conclusion, we report high infection rates among wild-caught vectors. Early stages of parasite infection up to the development of oocysts does not appear to influence survival for the two major vectors identified in this study. In contrast to *An. funestus*, *An. gambiae s.s*. exhibits increased mortality when collected from households with higher ITN coverage, although the mortality rate is still lower than would be expected. Rearing wild-caught mosquitoes gives unique insights into exposures correlated with vector survival and development of infectivity.

## Availability of data:

The datasets used and/or analyzed during the current study are available from the corresponding author on reasonable request.

## Figures and Tables

**Table 1:** Number and proportions of *Anopheles* species captured by indoor resting collection per village

	Village					
Species	K	L	M	N	S	Total
*An. gambiae s.s*.	81 (49.7%)	22 (42.3%)	69 (23.9%)	37 (43.5%)	97 (78.9%)	306 (42.9%)
*An. funestus*	55 (33.7%)	20 (38.5%)	171 (59.2%)	30 (35.3%)	7 (5.7%)	283 (39.8%)
*An. gambiae s.l*.^1^	11 (6.8%)	4 (7.7%)	25 (8.7%)	12 (14.1%)	7 (5.7%)	59 (8.3%)
*An. arabiensis*	8 (4.9%)	3 (5.8%)	0 (0.0%)	1 (1.2%)	3 (2.4%)	15 (2.1%)
*An. demeilloni*	0	2 (3.9%)	6 (2.1%)	0	2 (1.6%)	10 (1.4%)
*An. rufipes*	1 (0.6%)	0	6 (2.0%)	2 (2.4%)	0	9 (1.3%)
Undetermined	7 (4.3%)	1 (1.9%)	12 (4.1%)	3 (3.5%)	7 (5.7%)	30 (4.2%)
**Total**	**163**	**52**	**289**	**85**	**123**	**712**

**Table 2:** Abdominal *P. falciparum* infection by species

Mosquito Species	Total tested	Number infected	Oocyst rate per species
*An. gambiae s.s*.	298	18	6.0%
*An. funestus*	271	27	10.0%
*An. gambiae s.l*.	55	6	10.9%
Undetermined	29	2	6.9%
*An. arabiensis*	15	0	0
*An. demeilloni*	8	0	0
*An. rufipes*	7	0	0
Total	683	53	7.8%
